# A commentary on: “CD33 drives cutaneous melanoma: mendelian randomization confirms causality, multi-omics and in vitro experiments reveal M2 macrophage polarization-mediated progression”

**DOI:** 10.1097/JS9.0000000000004789

**Published:** 2026-01-21

**Authors:** Xiaoqi Sun, Lihui Xu, Suqin Ni

**Affiliations:** Department of Plastic Surgery, Affiliated Xiaoshan Hospital, Hangzhou Normal University, Hangzhou, China

*Dear Editor,*

With significant advances in immunotherapy and gene-targeted treatments for cutaneous melanoma (CM), metastatic melanoma can now be effectively treated[1]. Nevertheless, whether using single-agent or combination therapies, the majority of patients still fail to achieve sustained therapeutic responses. Moreover, the high recurrence rates and incidence of immune resistance underscore the fact that the treatment of metastatic melanoma remains a formidable challenge^[2,3]^. There is an urgent need for novel therapeutic targets and pathways to improve treatment outcomes and prognosis. Hu *et al* conducted multiple Mendelian randomization analyses and transcriptomic studies utilizing data from multiple databases. Through *in vitro* cellular validation, it was demonstrated that reducing CD33 expression on M2 macrophage surfaces inhibits melanoma cell proliferation and migration, thereby providing novel therapeutic insights for CM[4].This commentary adheres to the TITAN Guidelines 2025 for the declaration and use of artificial intelligence in scientific publications, and no AI tools were used in the design, analysis, or writing of this work[5].

This study employs a three-tiered strategy “population-based Mendelian genetics, single-cell multi-omics analysis, and in vitro functional validation.” First, bidirectional Mendelian randomization was performed using GWAS data with 731 immune cell traits and deCODE plasma pQTLs as instrumental variables. Subsequently, causal relationships were reinforced through SMR/HEIDI/co-localization Mendelian randomization analyses. Finally, the validation process was conducted by regressing towards single-cell multi-omics studies and *in vitro* molecular experiments. Research has revealed a positive causal relationship between CD33 and CM, primarily concentrated within M2 macrophages. Furthermore, CD33 has been shown to promote the proliferation of CM by activating the STAT3/STAT6 signaling pathway, polarizing M2 macrophages and ultimately reducing patient survival rates.

The data for this study were sourced from multiple databases, including Finngen, TCGA, GTEx, and GENT2, thus ensuring a substantial sample size. This also lends the results greater statistical significance. Furthermore, this study incorporated seven MR algorithms and conducted further validation through *in vitro* cell experiments, thereby establishing a more robust causal relationship between CD33 and CM.

However, this study still has some limitations. First, the pQTL data for the CD33 protein in this study were derived from peripheral blood plasma. Whether this correlates with CD33 expression in skin tissue remains unvalidated, and potential exposure measurement bias may exist. Second, M2 macrophages induced in THP-1 cells by PMA + IL-4/10/13 + dexamethasone represent only an “inflammatory M2c-like” phenotype and cannot fully replicate the heterogeneity of tumor-associated macrophages[6]. Moreover, only two CM cell lines – A375 and SK-MEL-28 – were included. Incorporating additional phenotypes such as NRAS mutations and NF1 deletions could enhance the study’s generalizability^[7,8]^. Finally, the sample data for this study were derived exclusively from European populations. Given that epidemiological patterns and immunotherapy responses for CM exhibit significant variations across different skin tones, incorporating data from East Asian, Latin American, and African populations would enhance the guidance value of the findings[9].

In general, the research conducted by HU *et al* has concentrated on the function of the CD33-M2 axis in CM, thus providing novel targets for research into the tumor microenvironment and tumor immunotherapy. Concurrently, the monoclonal antibody CD33 shows promise for future combination therapy with PD-1 inhibitors to overcome immune resistance, marking a crucial step forward in treating CM. Our suggestions are merely intended to further refine what is already an excellent piece of research, and we eagerly anticipate further innovative work from the authors in the future.

## Data Availability

No other datasets were generated and/or analyzed during the current study. All the information is available with the manuscript.
